# Monoclonal Antibodies versus Histone Deacetylase Inhibitors in Combination with Bortezomib or Lenalidomide plus Dexamethasone for the Treatment of Relapsed or Refractory Multiple Myeloma: An Indirect-Comparison Meta-Analysis of Randomized Controlled Trials

**DOI:** 10.1155/2018/7646913

**Published:** 2018-06-27

**Authors:** Yanhua Zheng, Hongyuan Shen, Li Xu, Juan Feng, Hailong Tang, Na Zhang, Xiequn Chen, Guangxun Gao

**Affiliations:** Department of Hematology, Xijing Hospital, Fourth Military Medical University, Xi'an, Shaanxi, China

## Abstract

During the past decades, agents with novel mechanisms of action, such as monoclonal antibodies (MAbs) and histone deacetylase inhibitors (HDACis) have been applied to treat relapsed or refractory multiple myeloma (RRMM). The treatment outcomes of MAbs versus HDACi in combination with bortezomib or lenalidomide plus dexamethasone remain unknown. We conducted this meta-analysis to compare indirectly the efficacy and safety of MAbs and HDACis in combination with bortezomib or lenalidomide plus dexamethasone. Six trials (eight articles) were included in the meta-analysis with 3270 RRMM patients enrolled. We synthesized hazard ratios (HRs) for progression-free survival (PFS) and overall survival (OS), risk ratios (RRs) for complete response (CR),very good partial response (VGPR), overall response (OR), progressive disease plus stable disease (PD + SD) and common at least grade 3 adverse events, and their corresponding 95%confidence intervals (95% CI). Treatment with MAbs in combination with bortezomib or lenalidomide plus dexamethasone resulted in longer PFS (HR 0.83, 95% CI: 0.66–0.98), fewer incidences of at least grade 3 thrombocytopenia (RR 0.35, 95% CI: 0.23–0.53), neutropenia (RR 0.70, 95% CI: 0.51–0.96), and sense of fatigue (RR 0.37, 95% CI: 0.17–0.82) than HDACis. The daratumumab plus bortezomib or lenalidomide and dexamethasone might significantly improve PFS in comparison with HDACis plus bortezomib or lenalidomide and dexamethasone (HR 0.55, 95% CI: 0.40–0.74). In conclusion, MAbs may be superior to HDACis in achieving longer PFS and may be better tolerated when in combination therapy with bortezomib or lenalidomide plus dexamethasone.

## 1. Introduction

Multiple myeloma (MM) is the second most commonly diagnosed hematological malignancy, characterized by the accumulation of high levels of monoclonal immunoglobulins in blood or urine, leading to anemia, hypercalcemia, renal dysfunction, and bone lesions [1]. During the past decades, significant prolongation of overall survival (OS) has been achieved with the incorporation of autologous stem cell transplantation (ASCT), proteasome inhibitors (PIs), and particularly bortezomib and immunomodulatory drugs (IMiDs), such as thalidomide and lenalidomide [2, 3]. Despite the advancements in the treatment, MM remains incurable because acquired or intrinsic resistance to therapy results in eventual relapse and the disease becomes refractory [4]. Therefore, novel agents with different mechanisms of action including monoclonal antibodies (MAbs) and histone deacetylase inhibitors (HDACis) have been developed as new treatment approaches for relapsed or refractory multiple myeloma (RRMM).

MAbs can induce tumor cell killing by targeting specific antigens expressed on MM cells through various mechanisms including antibody-dependent cell-mediated cytotoxicity (ADCC), antibody-dependent cellular phagocytosis (ADCP), complement-dependent cytotoxicity (CDC), and other direct effects such as alterations in intracellular signaling and inhibition of functions of adhesion molecules [5]. Daratumumab (IgG1-*κ*;fully human), isatuximab (IgG1-*κ*;chimeric), and MOR202 (IgG1-*λ*;fully human) are MAbs that target CD38, a transmembrane glycoprotein, which is highly and ubiquitously expressed on MM cells and at low degrees expressed on normal cells [6]. Elotuzumab is a humanized IgG1-*κ* immunostimulatory MAb targeting signaling lymphocytic activation molecule F7 (SLAMF7), also referred to as cell-surface glycoprotein CD2 subset1 (CS1) which is expressed on MM cells and natural killer cells. Elotuzumab exerts its antimyeloma effects by mediating ADCC, directly activating natural killer cells, and inhibiting the interactions between MM cells and stromal cells [7, 8].

There is preclinical evidence that overexpression of HDAC has been found in MM, while inhibition of HDAC leads to the blockade of aggresome and ubiquitin-proteasome pathways and increased acetylation of histone proteins, which regulate the expression of tumor suppressor genes and transcriptional factors, elucidating the synergistic antimyeloma effect of bortezomib and HDACi when used in combination [9, 10]. Panobinostat was approved by the US Food and Drug Administration (FDA) in February 2015 for the treatment of RRMM in combination therapy. Other HDACis include vorinostat, romidepsin, belinostat, and ricolinostat [11].

In recent years, several randomized controlled trials (RCTs) have been performed to evaluate the efficacy and safety of the novel agents combined with bortezomib or lenalidomide plus dexamethasone in RRMM patients. However, the treatment outcomes of MAbs versus HDACis in combination with bortezomib or lenalidomide plus dexamethasone remain enigmatic. We thus conducted this meta-analysis to compare indirectly the efficacy and safety of MAbs and HDACis in combination with lenalidomide or bortezomib plus dexamethasone.

## 2. Materials and Methods

This indirect-comparison meta-analysis was conducted in accordance with the quality of reporting of meta-analyses (QUOROM) statements [12].

### 2.1. Literature Retrieval Strategy

We searched for relevant studies in the database of Pubmed, Embase (OVID), The Cochrane Library, and Web of Science. The following medical subject headings (MeSH) or keywords were used in literature retrieval: “multiple myeloma” OR “myeloma,” “relapsed” OR “refractory,” “monoclonal antibodies” OR “daratumumab” OR “elotuzumab” OR “Isatuximab” OR “MOR202,” “histone deacetylase inhibitors” OR “HDACi” OR “panobinostat” OR “vorinostat” OR “romidepsin” OR “belinostat” OR “ricolinostat.” The language was restricted in English. The latest retrieval was updated on December 9, 2017.

### 2.2. Selection Criteria

To guarantee the reliability and verifiability of our analysis, all the eligible studies had to meet the following prespecified inclusion criteria: (1) they were RCTs no matter whether they adopted blinding or not; (2) the patients were diagnosed with relapsed or refractory multiple myeloma; (3) either placebo control or blank control was qualified in the control group; (4) they presented adequate information about the hazard ratios (HRs) and 95% confidence intervals (CIs) or Kaplan-Meier curves for at least one of the following survival endpoints: time to progression (TTP), progression-free survival (PFS), and overall survival (OS); (5) they provided the exact number of patients who achieved any grade status of response to treatment, including complete response (CR), very good partial response (VGPR), partial response (PR), stable disease (SD), progressive disease (PD); and (6) grade 3 or higher treatment-related adverse events were the safety outcomes including hematological toxicities and common nonhematological adverse events.

The exclusion criteria were nonhuman experiments, review articles, case reports, conference abstracts, duplicate publications, or other studies which are irrelevant to our topic and failed to provide sufficient information.

### 2.3. Study Qualitative Assessment and Data Extraction

Qualitative assessment for each included articles was conducted using the Jadad scale that was chiefly concerned with three aspects, including randomization method, double-blinding, and outcomes of follow-up [13]. Articles with Jadad score 3 to 5 were considered to be of high quality, while articles with Jadad score 1 to 2 were regarded as low-quality studies.

Two investigators independently extracted all the data (baseline characteristics, outcomes of survival analysis, treatment response, and adverse events) from the original articles. If there occurred discrepancies, they reached a consensus by discussion. Meanwhile, a third senior investigator inspected the process of data input.

In this meta-analysis, the primary efficacy endpoint was PFS which was calculated from the date of randomization to the time of disease progression, recurrence, or death due to any cause (whichever occurred first). One secondary efficacy endpoint was OS which was measured from the date of randomization until death from any cause or the last follow-up observation of patients. So HR value and corresponding 95% CI for PFS, OS, or TTP had to be extracted directly from the original articles through full-text screening. If a study did not provide HR value for PFS, OS, or TTP, we prepared to resort to Engauge Digitizer version 4.1 to distinguish survival curves and estimate HRs and 95% CI according to the methods published by Tierney et al. [14].

Other secondary efficacy endpoints were overall response rate (ORR), proportion of very good partial response (VGPR) or better (comprising very good partial, complete, and stringent complete response), and proportion of complete response (CR) or better (comprising complete and stringent complete response). In this meta-analysis, overall response was defined as at least partial response (comprising partial, very good partial, complete, and stringent complete responses), while progressive disease (PD) plus stable disease (SD) were considered ineffective treatment. So the number of patients who achieved OR, VGPR or better, CR or better, and PD plus SD after treatment both in the experimental and the controlled group required to be extracted.

Safety outcomes, including anemia, neutropenia, thrombocytopenia, and some common nonhematological events, were represented by the evaluation of common grade 3 or 4 adverse events according to the National Cancer Institute Common Terminology Criteria for Adverse Events, version 4.03. The number of patients in the two groups who suffered from those adverse events were also needed to be recorded in the predesigned tables.

### 2.4. Statistical Analysis

HRs with corresponding 95% CI for PFS, OS, or TTP were used to compare the survival outcomes. Risk ratios (RRs) with 95% CI were used to compare the treatment response status and adverse event incidence.

The heterogeneity among studies was quantified by means of chi-squared test (*χ*^2^ test) and *I*-squared test (*I*^2^ test). Any value of *I*^2^ less than 25% was defined low heterogeneity, and 25% to 50% was regarded intermediate. In case of low and intermediate heterogeneity, fixed-effects model (Mantel-Haenszel method) was used to generate the pooled HR for PFS or OS and the pooled RR for OR, VGPR, CR, and PD plus SD by subgroup analysis. If *I*^2^ was greater than 50%, the heterogeneity was considered statistically significant, and afterwards, analysis was conducted using random-effects model (Der Simonian-Liard method) to synthesize the effect value [15]. In order to explore the efficacy and safety of MAbs versus HDACis combined with bortezomib or lenalidomide plus dexamethasone in the treatment of RRMM, we intended to conduct an indirect-comparison meta-analysis between the two subgroups (MAb group and HDACi group). If the 95% CI did not overlap 1, the pooled HR or RR was considered to be statistically significant. Subgroup meta-analysis between either MAb group or HDACi group and their corresponding control group were also presented in the form of forest plot, but the results of indirect-comparison of MAb versus HDACi could not be shown in the form of forest plot.

Publication bias for each synthesized effect size was evaluated using Egger's test and Begg's test. If *P* value was less than 0.05, significant publication bias existed.

We employed Stata statistical software version 12.0 (Stata Corporation, College Station, TX, USA) to perform all the meta-analysis and tested publication bias. The indirect comparison procedures of MAb group versus HDACi group were conducted using the Stata indirect program package. All tests were two sided with *P* < 0.05 considered statistically significant.

## 3. Results

### 3.1. Literature Search Results and Study Characteristics

**Figure 1:**
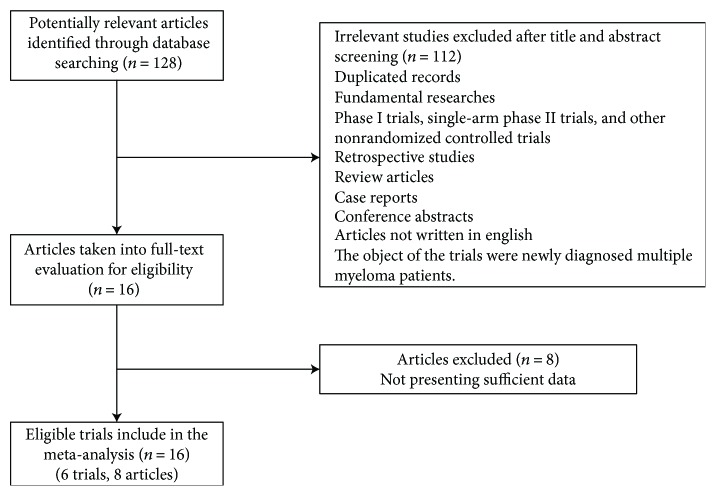
Identification and selection process of clinical trials included in the meta-analysis.

The flowchart of the literature selection process was presented in Figure 1. A total of 128 articles were identified through database searching. After meticulously inspecting the titles and abstracts, 112 articles were eliminated because of duplicate records, fundamental researches, retrospective studies, case reports, and phase I and single-arm phase II trials. Among the remaining 16 articles, 8 articles were excluded through full-text screening because they failed to present sufficient outcome data. Eventually, our meta-analysis included altogether 6 RCTs (8 articles) with a total of 3270 patients enrolled [16–23]. Four RCTs explored the efficacy and safety of the use of MAbs (including daratumumab and elotuzumab) or not combined with bortezomib or lenalidomide plus dexamethasone in patients with RRMM. The other two RCTs compared HDACis (including panobinostat and vorinostat) versus placebo in combination with bortezomib plus dexamethasone in RRMM patients. The main outcomes of included articles were summarized in Table 1. The demographic baseline and patients' characteristics of each RCT were well balanced between the two groups and then summarized in Table 2.

According to Jadad scale [13], we roughly assessed six included trials, all of which were considered high quality. Four RCTs had Jadad score of 3, and the other two RCTs had Jadad score of 5. The detailed information of study quality assessment was demonstrated in Table 3.

### 3.2. Efficacy

The meta-analysis outcomes of efficacy were summarized in Table 4 and Table 5.

#### 3.2.1. Progression-Free Survival

**Figure 2:**
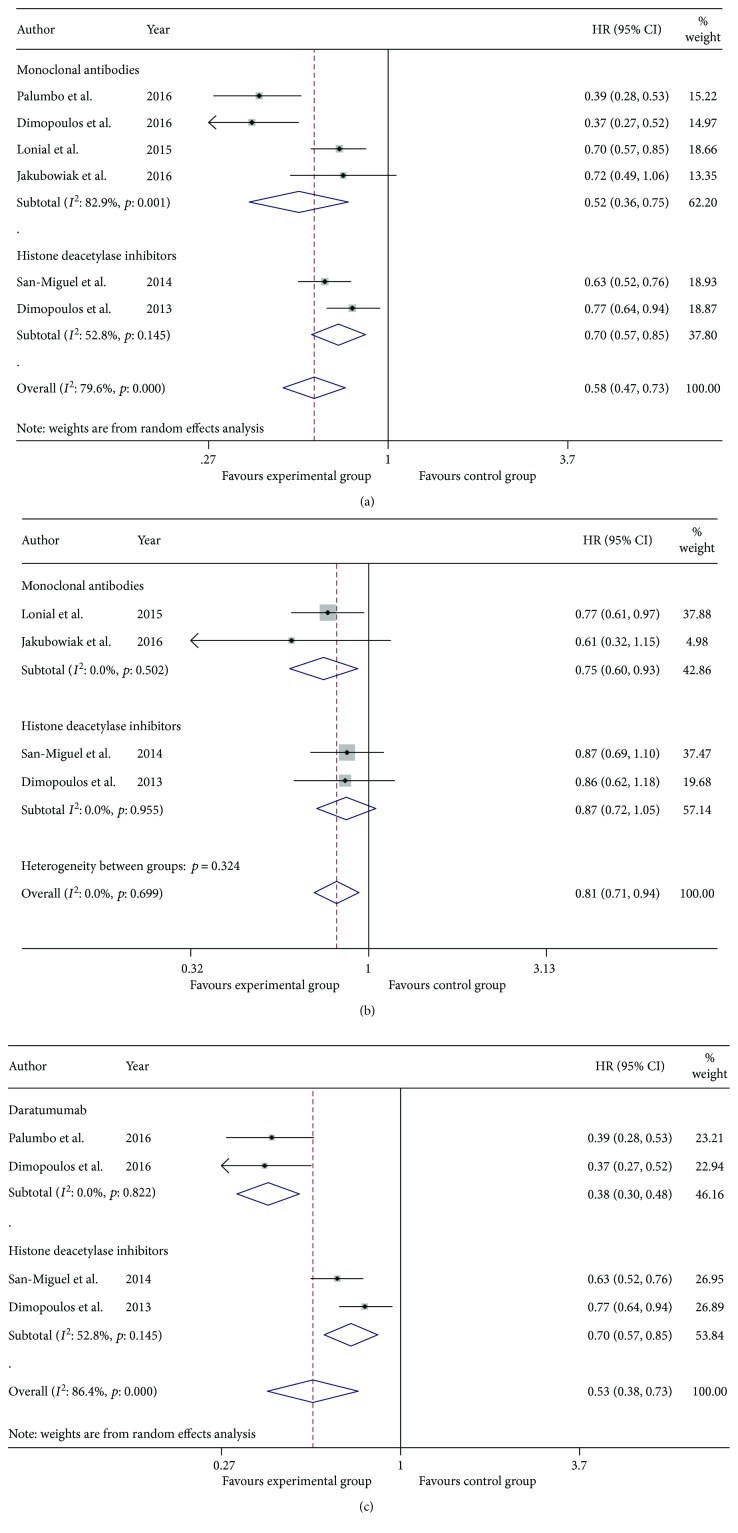
Meta-analysis of the efficacy of MAb group and HDACi group in patients with RRMM: (a) hazard ratio for progression free survival of MAb group and HDACi group versus their corresponding control group; (b) hazard ratio for overall survival of MAb group and HDACi group versus their corresponding control group; and (c) hazard ratio for progression-free survival of daratumumab group and HDACi group versus their corresponding control group. “*P* = 0.000” in Figure 2(a) and Figure 2(c), which was automatically generated by Stata software, represents *P* ≤ 0.001 actually, denoting that there exists heterogeneity among studies.

Data on PFS were available in all of the six included studies. We performed a subgroup analysis and indirect comparison of MAbs and HDACis in combination with bortezomib or lenalidomide plus dexamethasone. As was illustrated in Figure 2(a), the pooled HR of MAbs group versus control group was 0.52 (95% CI 0.36–0.75), and the pooled HR of HDACi group versus control group was 0.70 (95% CI 0.57–0.85). The indirect-comparison results indicated that MAb group might improve PFS when compared with HDACi group, yielding pooled HR 0.83 (95% CI 0.66–0.98), which represented a 17% lower risk of progression or death in the MAb group than in the HDACi group. We safely came to the conclusion that the addition of either MAbs or HDACi to bortezomib or lenalidomide plus dexamethasone could significantly lengthen PFS in comparison with bortezomib or lenalidomide plus dexamethasone alone. We could also deduce that PFS was longer in the MAb group than in the HDACi group through indirect-comparison meta-analysis.

After removing all the trials about elotuzumab, we then did the same analysis procedure. As was illustrated in Figure 2(c), the pooled HR of daratumumab group versus control group was 0.38 (95% CI 0.30–0.48) and that of HDACi group versus control group was 0.70 (95% CI 0.57–0.85). The indirect-comparison results showed that daratumumab group might significantly improve PFS in comparison with HDACi group, yielding pooled HR 0.55 (95% CI 0.40–0.74), which suggested that daratumumab group dramatically reduced the risk of disease progression or death by 45% compared with HDACi group. We could infer from the abovementioned that daratumumab in combination with bortezomib or lenalidomide plus dexamethasone might significantly prolong PFS when compared with HDACi in combination with bortezomib or lenalidomide plus dexamethasone.

#### 3.2.2. Overall Survival

Because two included trials about daratumumab failed to provide adequate OS data, we conducted a subgroup analysis and indirect comparison of elotuzumab and HDACis in combination with bortezomib or lenalidomide plus dexamethasone. As was shown in Figure 2(b), the pooled HR of elotuzumab group versus control group was 0.75 (95% CI 0.60–0.93) and that of HDACi group versus control group was 0.87 (95% CI 0.72–1.05). The indirect-comparison results generated pooled HR of 0.87 (95% CI 0.65–1.15), suggesting that the elotuzumab group did not gain an advantage over the HDACi group on OS.

#### 3.2.3. OR, VGPR, CR, and PD plus SD

**Figure 3:**
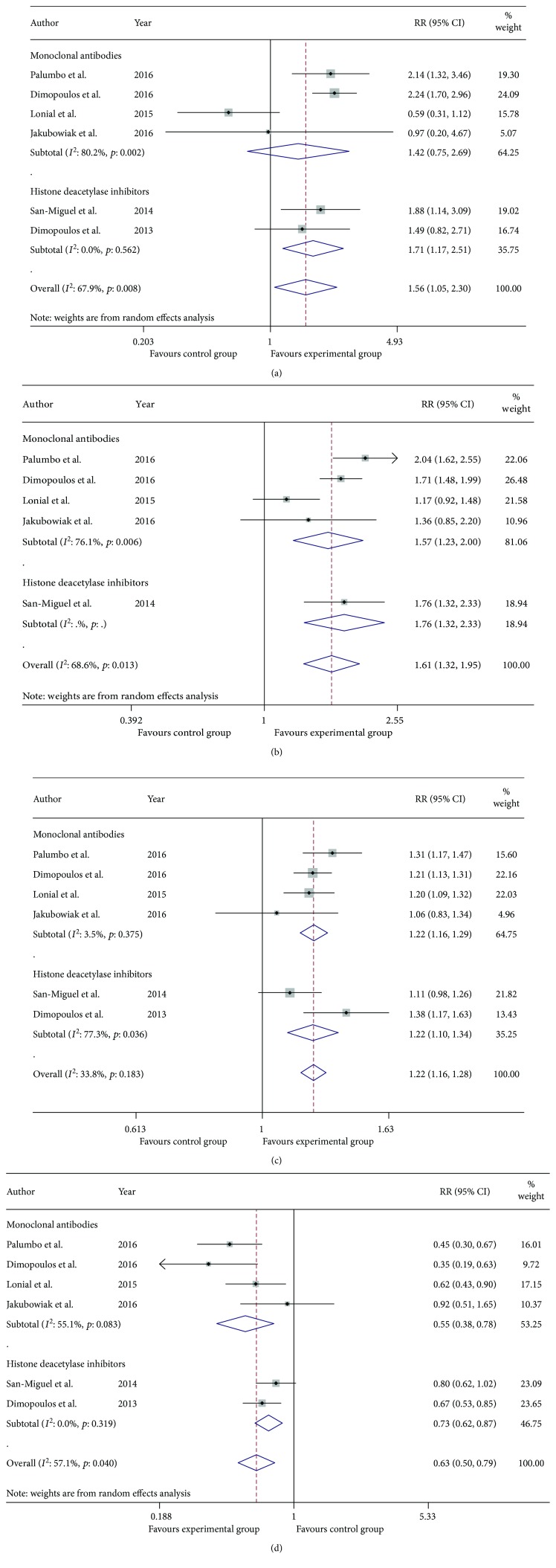
Meta-analysis of the efficacy of MAbs group and HDACi group in patients with RRMM: risk ratio for complete response (a), very good partial response (b), overall response (c), and progressive disease plus stable disease (d) of MAb group and HDACi group versus their corresponding control group.

**Figure 4:**
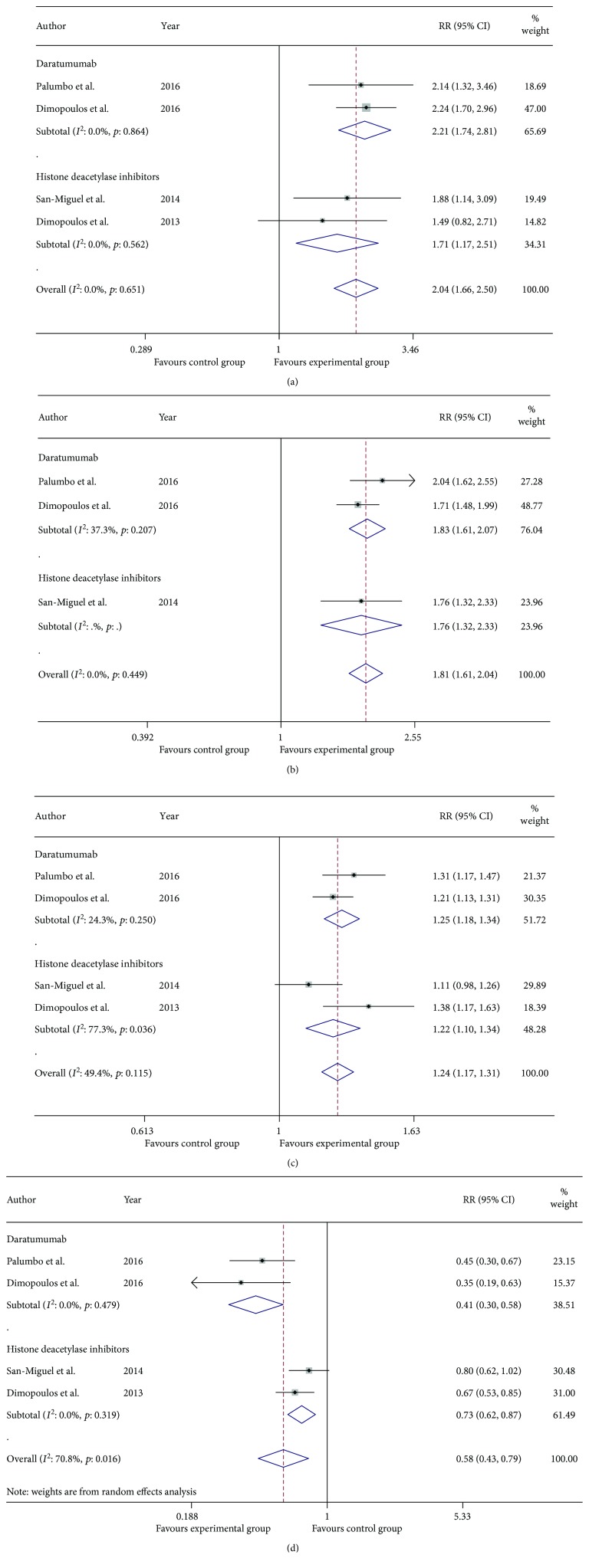
Meta-analysis of the efficacy of daratumumab group and HDACi group in patients with RRMM: risk ratio for complete response (a), very good partial response (b), overall response (c), and progressive disease plus stable disease (d) of daratumumab group and HDACi group versus their corresponding control group.

As was shown in Figure 3, the pooled RRs for OR, VGPR, CR, and PD plus SD in the MAb group versus control group were 1.22 (95% CI 1.16–1.29), 1.57 (95% CI 1.23–2.00), 1.42 (95% CI 0.75–2.69), and 0.55 (95% CI 0.38–0.78), respectively. Likewise, the pooled RRs for OR, VGPR, CR, and PD plus SD in the HDACi group versus control group were 1.22 (95% CI 1.10–1.34), 1.76 (95% CI 1.32–2.33), 1.71 (95% CI 1.17–2.51), and 0.73 (95% CI 0.62–0.87), respectively. The indirect comparison of MAb group versus HDACi group generated pooled RR 1.04 (95% CI 0.91–1.18) for OR, 0.83 (95% CI 0.44–1.57) for VGPR, 0.85 (95% CI 0.23–3.12) for CR, and 0.80 (95% CI 0.65–0.94) for PD plus SD. In summary, we concluded that both MAb group and HDACi group were superior to their corresponding control groups while there was no evidence showing that MAbs exhibited more benefits than HDACis when combined with bortezomib or lenalidomide plus dexamethasone concerning OR, VGPR, and CR. In terms of PD plus SD (previously defined as ineffective treatment), both MAbs group and HDACis group acted as protective factors from the ineffective treatment when compared with their corresponding control groups. The indirect comparison showed that MAbs served as stronger protective factors than HDACis against invalid therapy, which indicated that MAbs might be more effective than HDACis. After omitting trials about elotuzumab, the conclusions derived from meta-analysis outcomes shown in Figure 4 and Table 5 were that daratumumab served as stronger protective factors than HDACis against invalid treatment.

### 3.3. Safety

Table 6 demonstrated the meta-analysis outcomes of common at least grade 3 hematological and nonhematological adverse events.

**Figure 5:**
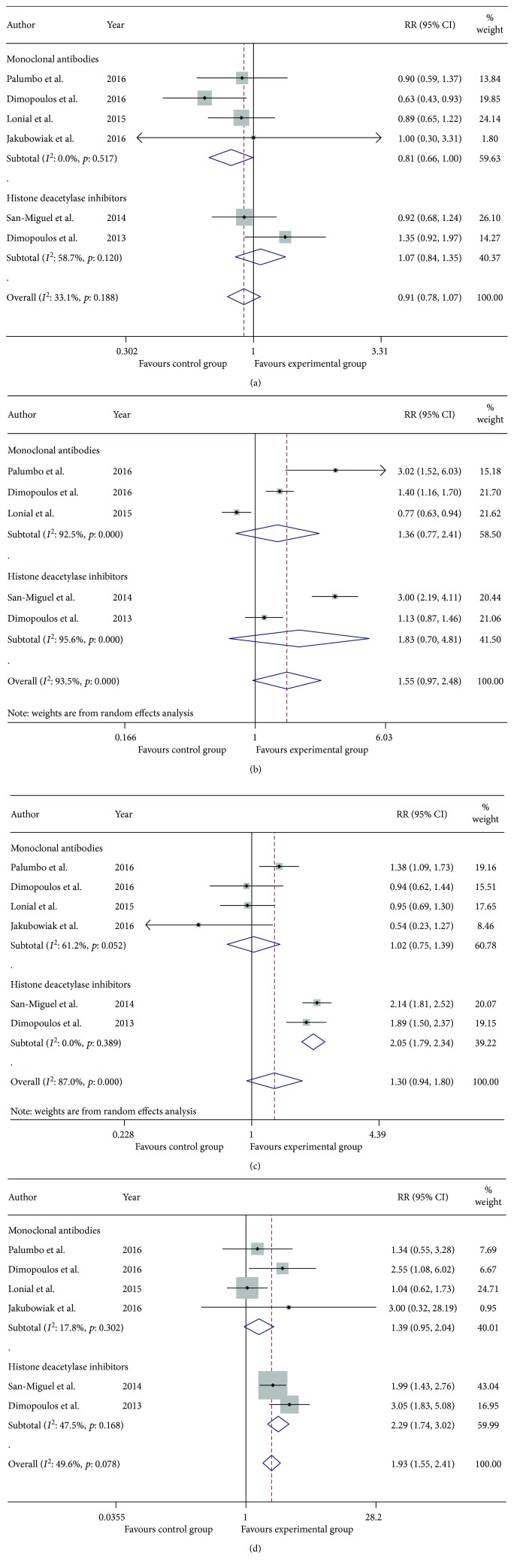
Meta-analysis of the safety of MAbs group and HDACi group in patients with RRMM: risk ratio for at least grade 3 anemia (a), neutropenia (b), thrombocytopenia (c), and sense of fatigue (d) of MAb group and HDACi group versus their corresponding control group. “*P* = 0.000” in Figure 5(b) and Figure 5(c), which was automatically generated by Stata software, represents *p* ≤ 0.001 actually, denoting that there exists heterogeneity among studies.

Figure 5 showed risk ratios of MAb group and HDACi group versus their corresponding control group for at least grade 3 anemia, neutropenia, thrombocytopenia, and sense of fatigue. There were more incidences of at least grade 3 diarrhea (RR 1.63, 95% CI: 1.03–2.58) in the MAb group than in the control group. There were more incidences of at least grade 3 thrombocytopenia (RR 2.05, 95% CI: 1.79–2.34), upper respiratory tract infection (RR 2.56, 95% CI: 1.08–6.07), sense of fatigue (RR 2.29, 95% CI: 1.74–3.02), and diarrhea (RR 2.56, 95% CI: 1.93–3.41) in the HDACi group than in the control group.

After indirect-comparison meta-analysis, there were fewer incidences of at least grade 3 neutropenia (RR 0.70, 95% CI: 0.51–0.96) and thrombocytopenia (RR 0.35, 95% CI: 0.23–0.53) in the MAb group than in the HDACi group when combined with bortezomib or lenalidomide plus dexamethasone, while equivalent frequencies of at least grade 3 anemia (RR 0.79, 95% CI: 0.59–1.07) were observed between the two groups. With respect to nonhematological adverse events, fewer incidences of at least grade 3 sense of fatigue were found in the MAb group than in the HDACi group, giving RR 0.37 (95% CI, 0.17–0.82). There existed no significant differences between the MAb group and the HDACi group in the incidence of other common grade 3 or 4 adverse events such as nausea or vomiting, peripheral neuropathy, pyrexia, constipation, diarrhea, and upper respiratory tract infection.

### 3.4. Publication Bias

As indicated in Table 4, Table 5, and Table 6, there were no evident publication bias for all the meta-analysis outcomes by Egger's test and Begg's test.

## 4. Discussion

With the application of proteasome inhibitors and immunomodulatory drugs in the treatment of MM, the survival outcomes have significantly improved. However, most patients will inevitably have a relapse even after complete response and then the disease becomes refractory to treatment [24]. So there is an urgent need for addition of agents with novel mechanisms of action to conventional therapy regimen in an attempt to explore more effective and better-tolerated combination regimens.

Several preclinical studies have demonstrated that both bortezomib and lenalidomide can augment the antimyeloma effect of CD38-targeting antibodies and elotuzumab [25–27], thus forming the rationale for the clinical assessment of the combination therapy of a MAb plus bortezomib or lenalidomide. Meanwhile, combination regimen with HDACi and proteasome inhibitors or immunomodulatory drugs shows remarkable antimyeloma effect in both preclinical and clinical background [28, 29]. Recently, an extended 3-year follow-up report of ELOQUENT-2 and post hoc analyses shows that 1-, 2-, and 3-year OS rates with elotuzumab group versus control group were 91% versus 83%, 73% versus 69%, and 60% versus 53%, respectively. Besides, by means of serum M-protein dynamic modeling, the elotuzumab group exhibited a slower tumor regrowth than the control group [17]. A network meta-analysis, which includes 18 treatment options for RRMM, shows that the combination regimen of daratumumab, lenalidomide, and dexamethasone (POLLUX) seems to be the best option according to the synthesized HR for PFS, but HR for OS and adverse events were not discussed [30]. Up till now, no comparison has been made between MAb and HDACi in combination with bortezomib or lenalidomide plus dexamethasone.

To the best of our knowledge, this is the first meta-analysis of prospective RCTs designed to explore the efficacy and safety of MAbs versus HDACis combined with bortezomib or lenalidomide plus dexamethasone in RRMM patients. Among patients with RRMM, the combination of a kind of MAb, bortezomib or lenalidomide and dexamethasone might result in longer progression-free survival than the combination of a kind of HDACi, bortezomib or lenalidomide and dexamethasone, with a pooled HR for PFS of 0.83 (95% CI: 0.66–0.98), indicating a relative reduction of 17% in the risk of disease progression or death. After removing trials regarding elotuzumab, we acquired a synthesized HR for PFS of HR 0.55 (95% CI: 0.40–0.74), which represented a 45% lower risk of progression or death in the daratumumab group than the HDACi group. As compared with the HDACi group, overall survival was not prolonged in the MAb group with a HR for OS of 0.87 (95% CI: 0.65–1.15). Meanwhile, complete response, very good partial response, and overall response showed no statistical significance between the MAb group and the HDACi group.

As for toxicities, there were fewer incidences of at least grade 3 neutropenia (RR 0.70, 95% CI: 0.51–0.96) and thrombocytopenia (RR 0.35, 95% CI: 0.23–0.53) in the MAb group than in the HDACi group, while equivalent frequencies of at least grade 3 anemia (RR 0.79, 95% CI: 0.59–1.07) were observed between the two groups. We did not take infusion-related reactions of MAbs into consideration in this meta-analysis. The reasons are as follows. Infusion-related reactions mainly occurred during the first infusion, which were predominantly restricted to grade 1 to 2 events characterized by pyrexia, chills, vomiting, rash, cough, transient dyspnea, and hypertension. A meta-analysis showed that no matter which kind of MAbs was used, either as a single agent or in combination therapy, the proportion of patients who discontinued therapy due to infusion-related reactions was very low [31]. When a patient suffers from infusion-related reactions, the infusion should be temporarily suspended and dexamethasone and antihistamines can be administered as a method of treatment or prophylaxis [32].

In recent years, therapeutic blockades of immune checkpoint pathways, particularly programmed death 1 (PD-1), PD ligand 1 (PD-L1), and cytotoxic T-lymphocyte associated protein 4 (CTLA-4), have become research hotspots in various hematological malignancies [33]. Preclinical studies have revealed that MM cells can express PD-L1, which results in attenuated effect of cytotoxic T cell killing [34]. Besides, lenalidomide can enhance PD-1/PD-L1 blockade-induced antimyeloma effect, providing the theoretical evidence for combination therapy of immunomodulatory drugs and immune checkpoint inhibitors [35]. Currently, there are several ongoing and upcoming clinical trials evaluating the combined application of immune checkpoint blockading agents such as anti-PD-1/PD-L1 antibodies and anti-CTLA4 antibodies with other antimyeloma drugs in RRMM patients [36], which may provide more effective combination regimens in the near future.

There existed some limitations in our meta-analysis. Firstly, the synthesized calculation of all effect sizes in our meta-analysis was based on data that had already been published instead of primary-source individual patient data. Therefore, we could not carry out log-rank test and could not draw the Kaplan-Meier survival curves of PFS and OS among patients in the intention-to-treat population. Besides, HRs for PFS and OS could not be pooled by subgroups of age, gender, ISS disease staging, types of monoclonal immunoglobulin, cytogenetic abnormality risk profile, number of previous lines of treatment, previous treatment with autologous stem cell transplantation, and so on. Secondly, as with any meta-analysis, some baseline characteristics of patients were widely divergent among the trials. Furthermore, the duration of follow-up varied among the included trials. To some extent, the conclusion might be inaccurate because follow-up data regarding OS were still immature at the time of data cut-off, and HRs for OS or PFS were calculated at different follow-up durations [37]. So, longer follow-up data for OS are still needed. Thirdly, the included trials had excluded RRMM patents with severe hepatic, renal dysfunction, and poor performance status. The outcome of meta-analysis might not fully reflect real-word evidence and might not apply to patients with poor conditions. Fourthly, we did not perform the sensitivity analysis, which was designed to evaluate whether individual study could significantly influence the pooled results by removing each single study sequentially. Finally, although significant publication bias for each synthesized results were not detected using the Egger's test and Begg's test, we could not rule out the possibility of undetected publication bias due to the limited number of studies included.

In conclusion, our meta-analysis indicated that among patients with RRMM, the regimen of MAb in combination with bortezomib or lenalidomide plus dexamethasone was associated with longer PFS (HR 0.83), lower incidence of PD plus SD, and lower incidence of at least grade 3 thrombocytopenia, neutropenia and sense of fatigue as compared with HDACi in combination with bortezomib or lenalidomide plus dexamethasone. In other words, MAb is superior to HDACi when combined with bortezomib or lenalidomide plus dexamethasone from perspectives of both efficacy and safety. However, it remains still pivotal to conduct randomized controlled phase III trials to acquire head-to-head comparison evidence, further validating our findings.

## Figures and Tables

**Table 1:** Baseline characteristics of the included studies.

Study/reference	Phase	Number of patients	Treatment regimens	Median follow-up (months)	Primary endpoint	Median PFS (months)	1-year PFS rate (%)	Median OS (months)	1-year OS rate (%)
Palumbo et al. (2016) (CASTOR)	III	498	E: daratumumab 16 mg/kg + bortezomib 1.3 mg/m2 + dexamethasone 20 mg C: bortezomib 1.3 mg/m2 + dexamethasone 20 mg	E: 7.4 C: 7.4	PFS	E: NA C: 7.2	E: 60.7% C: 26.9%	E: NA C: NA	E: NA C: NA

Dimopoulos et al. (2016) (POLLUX)	III	569	E: daratumumab 16 mg/kg + lenalidomide 25 mg + dexamethasone 40 mg C: lenalidomide 25 mg + dexamethasone 40 mg	E: 13.5 C: 13.5	PFS	E: NA C: 18.4	E: 83.2% C: 60.1%	E: NA C: NA	E: 92.1% C: 86.8%

Lonial et al. (2015) (ELOQUENT-2) Dimopoulos et al. (2017) (ELOQUENT-2 follow-up)	III	646	E: elotuzumab 10 mg/kg + lenalidomide 25 mg + dexamethasone 40 mg C: lenalidomide 25 mg + dexamethasone 40 mg	E: 24.5 C: 24.5	PFS	E: 19.4 C: 14.9	E: 1-year PFS rate 68%; 2-year PFS rate 41% C: 1-year PFS rate 57% 2-year PFS rate 27%	E: 43.7 C: 39.6	E: 1-year OS rate 91%; 2-year OS rate 73% C: 1-year OS rate 83% 2-year OS rate 69%

Jakubowiak et al. (2016) (NCT01478048)	II	152	E: elotuzumab 10 mg/kg + bortezomib 1.3 mg/m2 + dexamethasone 20 mg C: bortezomib 1.3 mg/m2 + dexamethasone 20 mg	E: 15.9 C: 11.7	PFS	E: 9.7 C: 6.9	E: 1-year PFS rate 39%; 2-year PFS rate 18% C: 1-year PFS rate 33% 2-year PFS rate 11%	E: NA C: NA	E: 1-year OS rate 85%; 2-year OS rate 73% C: 1-year OS rate 74% 2-year OS rate 66%

San-Miguel et al. (2014) (PANORAMA1) San-Miguel et al. (2016) (PANORAMA1 follow-up)	III	768	E: panobinostat 20 mg + bortezomib 1.3 mg/m2 + dexamethasone 20 mg C: placebo + bortezomib 1.3 mg/m2 + dexamethasone 20 mg	E: 6.47 C: 5.59	PFS	E: 11.99 C: 8.08	E: 2-year PFS rate 20.6% C: 2-year PFS rate 8.4%	E: 40.3 C: 35.8	E: NA C: NA

Dimopoulos et al. (2013) (VANTAGE088)	III	637	E: vorinostat 400 mg + bortezomib 1.3 mg/m2 C: placebo + bortezomib 1.3 mg/m2	E: 14.2 C: 14.2	PFS	E: 7.63 C: 6.83	E: NA C: NA	E: NA C: 28.07	E: NA C: NA

PFS: progression-free survival; OS: overall survival; E: experimental group; C: control group; NA: not available.

**Table 2:** Patients' baseline characteristics and disease-related demographics of included studies.

Study	CASTOR		POLLUX		ELOQUENT-2		NCT01478048		PANORAMA1		VANTAGE088	
	E	C	E	C	E	C	E	C	E	C	E	C
Number of patients	251	247	286	283	321	325	77	75	387	381	317	320

Median age (year)	64	64	65	65	67	66	65	65	63	63	61	63

ECOG performance status												
0	NA	NA	139 (48.6)	150 (53.0)	NA	NA	38 (49.4)	46 (61.3)	175 (45.2)	162 (42.5)	126 (39.7)	119 (37.2)
1	NA	NA	1 o r2: 147 (51.4)	1 or 2: 133 (47.0)	NA	NA	35 (45.5)	23 (30.7)	191 (49.4)	186 (48.8)	164 (51.7)	167 (52.2)
2	NA	NA			NA	NA	2 (0.03)	6 (8.0)	19 (4.9)	29 (7.6)	24 (7.6)	34 (10.6)

ISS disease staging												
I	98 (39.0)	96 (38.9)	137 (47.9)	140 (49.5)	141 (43.9)	138 (42.5)	26 (33.8)	19 (25.3)	156 (40.3)	152 (39.9)	95 (30.0)	80 (25.0)
II	94 (37.5)	100 (40.5)	93 (32.5)	86 (30.4)	102 (31.8)	105 (32.3)	23 (29.9)	20 (26.7)	104 (26.9)	92 (24.1)	98 (30.9)	99 (30.9)
III	59 (23.5)	51 (20.6)	56 (19.6)	57 (20.1)	66 (20.6)	68 (20.9)	11 (14.3)	16 (21.3)	77 (19.9)	86 (22.6)	87 (27.4)	82 (25.6)
Not assessed					12 (0.04)	14 (0.04)	17 (22.1)	20 (26.7)	50 (12.9)	51 (13.4)	37 (11.7)	59 (18.4)
Previous lines of therapy												
1	122 (48.6)	113 (45.7)	149 (52.1)	146 (51.6)	151 (47.0)	159 (48.9)	50 (64.9)	51 (68.0)	197 (50.9)	198 (52.0)	143 (45.1)	127 (39.7)
2	70 (27.9)	74 (30.0)	85 (29.7)	80 (28.3)	118 (36.8)	114 (35.1)	2 or more: 27 (35.1)	2 or more: 24 (32.0)	124 (32.0)	108 (28.3)	105 (33.1)	134 (41.9)
3 or more	59 (23.5)	60 (24.3)	52 (18.2)	57 (20.1)	52 (16.2)	52 (16.0)			64 (16.5)	75 (19.7)	69 (21.8)	59 (18.4)

Previous stem-cell transplantation												
Yes	156 (62.2)	149 (60.3)	180 (62.9)	180 (63.6)	167 (52.0)	185 (56.9)	39 (50.6)	41 (54.7)	215 (55.6)	224 (58.8)	113 (35.6)	115 (35.9)
No	95 (37.8)	98 (39.7)	106 (37.1)	103 (36.4)	154 (48.0)	140 (43.1)	38 (49.4)	34 (45.3)	172 (44.4)	157 (41.2)	204 (64.4)	205 (64.1)

Drugs used in previous treatment												
Proteasome inhibitors	179 (71.3)	198 (80.2)	245 (85.7)	242 (85.5)	219 (68.2)	231 (71.1)	39 (50.6)	40 (53.3)	169 (43.7)	161 (42.3)	79 (24.9)	73 (22.8)
Immunomodulatory drugs	169 (67.3)	172 (69.6)	158 (55.2)	156 (55.1)	169 (52.6)	178 (54.8)	55 (71.4)	58 (77.3)	277 (71.6)	273 (71.7)	192 (60.6)	208 (65.0)
Alkylating agents	240 (95.6)	224 (90.7)	268 (93.7)	270 (95.4)	220 (68.5)	197 (60.6)	NA	NA	300 (77.5)	268 (70.3)	NA	NA

NA: not available; E: experimental group; C: control group.

**Table 3:** Quality assessment of included studies according to Jadad scale.

Study	Randomization	Blinding	Withdrawal or lost to follow-up	Total Jadad score
Palumbo et al. (2016)	2	0	1	3
Dimopoulos et al. (2016)	2	0	1	3
Lonial et al. (2015)	2	0	1	3
Jakubowiak et al. (2016)	2	0	1	3
San-Miguel et al. (2014)	2	2	1	5
Dimopoulos et al. (2013)	2	2	1	5

**Table 4:** Meta-analysis outcome of efficacy comparing monoclonal antibodies and HDACi.

	Number of trials included	Risk ratio (95% CI)			Tests for publication bias	
		MAb group versus control group	HDACi group versus control group	MAb group versus HDACi group (indirect comparison)	Egger's test (*P* value)	Begg's test (*P* value)
PFS	6 (trials 1, 2, 3, 4, 5, and 6)	HR 0.52 (0.36–0.75)	HR 0.70 (0.57–0.85)	HR 0.83 (0.66–0.98)	0.18	0.45
OS	4 (trials 3, 4, 5, and 6)	HR 0.75 (0.60–0.93)	HR 0.87 (0.72–1.05)	HR 0.87 (0.65–1.15)	0.39	0.73
CR	6 (trials 1, 2, 3, 4, 5, and 6)	1.42 (0.75–2.69)	1.71 (1.17–2.51)	0.85 (0.23–3.12)	0.17	0.02
VGPR	5 (trials 1, 2, 3, 4, and 5)	1.57 (1.23–2.00)	1.76 (1.32–2.33)	0.83 (0.44–1.57)	0.67	0.46
OR	6 (trials 1, 2, 3, 4, 5, and 6)	1.22 (1.16–1.29)	1.22 (1.10–1.34)	1.04(0.91–1.18)	0.89	1.00
PD + SD	6 (trials 1, 2, 3, 4, 5, and 6)	0.55 (0.38–0.78)	0.73 (0.62–0.87)	0.80 (0.65–0.94)	0.32	0.26

MAb: monoclonal antibody; HDACi: histone deacetylase inhibitor; PFS: progression-free survival; OS: overall survival; OR: overall response; CR: complete response; VGPR: very good partial response; PR: partial response; SD: stable disease; PD: progressive disease; HR: hazard ratio. Trials included: trial 1 represents Palumbo et al. (2016); trial 2 represents Dimopoulos et al. (2016); trial 3 represents Lonial et al. (2015); trial 4 represents Jakubowiak et al. (2016); trial 5 represents San-Miguel et al. (2014); trial 6 represents Dimopoulos et al. (2013).

**Table 5:** Meta-analysis outcome of efficacy comparing daratumumab and HDACi.

	Number of trials included	Risk ratio (95% CI)			Tests for publication bias	
		Daratumumab group versus control group	HDACi group versus control group	Daratumumab group versus HDACi group (indirect comparison)	Egger's test (*P* value)	Begg's test (*P* value)
PFS	4 (trials 1, 2, 5, and 6)	HR 0.38 (0.30–0.48)	HR 0.70 (0.57–0.85)	HR 0.55 (0.40–0.74)	0.06	0.31
CR	4 (trials1, 2, 5, and 6)	2.21 (1.74–2.81)	1.71 (1.17–2.51)	1.71 (0.72–4.06)	0.15	0.09
VGPR	3 (trials 1, 2, and 5)	1.83 (1.61–2.07)	1.76 (1.32–2.33)	1.03 (0.60–1.79)	0.66	1.00
ORR	4 (trials1, 2, 5, and 6)	1.25 (1.18–1.34)	1.22 (1.10–1.34)	1.06 (0.92–1.22)	0.64	0.73
PD + SD	4 (trials 1, 2, 5, and 6)	0.41 (0.30–0.58)	0.73 (0.62–0.87)	0.73 (0.60–0.88)	0.31	0.09

HDACi: histone deacetylase inhibitor; PFS: progression-free survival; OS: overall survival; ORR: overall response rate; CR: complete response; VGPR: very good partial response; PR: partial response; SD: stable disease; PD: progressive disease; HR: hazard ratio. Trials included: trial 1 represents Palumbo et al. (2016); trial 2 represents Dimopoulos et al. (2016); trial 3 represents Lonial et al (2015); trial 4 represents Jakubowiak et al. (2016); trial 5 represents San-Miguel et al. (2014); trial 6 represents Dimopoulos et al. (2013).

**Table 6:** Meta-analysis outcome of common at least grade 3 adverse events comparing monoclonal antibodies versus HDACis.

	Number of trials included	Risk ratio (95% CI)			Tests for publication bias	
		MAb group versus control group	HDACi group versus control group	MAb group versus HDACi group (indirect comparison)	Egger's test (*P* value)	Begg's test (*P* value)
Hematological adverse events						
Anemia	6 (trials 1, 2, 3, 4, 5, and 6)	0.81 (0.66–1.00)	1.07 (0.84–1.35)	0.79 (0.59–1.07)	0.95	1.00
Neutropenia	5 (trials 1, 2, 3, 5, and 6)	1.36 (0.77–2.41)	1.83 (0.70–4.81)	0.70 (0.51–0.96)	0.30	0.46
Thrombocytopenia	6 (trials 1, 2, 3, 4, 5, and 6)	1.02 (0.75–1.39)	2.05 (1.79–2.34)	0.35 (0.23–0.53)	0.03	0.26
Nonhematological adverse events						
Nausea or vomiting	4 (trials 2, 4, 5, and 6)	2.57 (0.66–9.99)	3.43 (0.91–12.91)	0.28 (0–398.63)	0.76	1.00
Peripheral neuropathy	4 (trials 1, 4, 5, and 6)	0.71 (0.40–1.27)	1.16 (0.85–1.58)	0.63 (0.35–1.14)	0.14	1.00
Upper respiratory tract infection	4 (trials 1, 2, 5, and 6)	1.38 (0.44–4.32)	2.56 (1.08–6.07)	0.71 (0.04–11.47)	0.31	0.40
Pyrexia	6 (trials 1, 2, 3, 4, 5, and 6)	0.89 (0.46–1.70)	0.91 (0.39–2.12)	1.02 (0.32–3.22)	0.47	1.00
Fatigue	6 (trials 1, 2, 3, 4, 5, and 6)	1.39 (0.95–2.04)	2.29 (1.74–3.02)	0.37 (0.17–0.82)	0.97	1.00
Constipation	6 (trials 1, 2, 3, 4, 5, and 6)	1.49 (0.53–4.16)	1.43 (0.55–3.73)	0.70 (0.05–10.53)	0.78	0.71
Diarrhea	6 (trials 1, 2, 3, 4, 5, and 6)	1.63 (1.03–2.58)	2.56 (1.93–3.41)	0.42 (0.15–1.19)	0.47	1.00

MAb: monoclonal antibody; HDACi: histone deacetylase inhibitor. Trials included: trial 1 represents Palumbo et al. (2016); trial 2 represents Dimopoulos et al. (2016); trial 3 represents Lonial et al. (2015); trial 4 represents Jakubowiak et al. (2016); trial 5 represents San-Miguel et al. (2014); trial 6 represents Dimopoulos et al. (2013).
